# Engineering Heterologous Production of Salicylate Glucoside and Glycosylated Variants

**DOI:** 10.3389/fmicb.2018.02241

**Published:** 2018-09-20

**Authors:** Ruiquan Qi, Blaine A. Pfeifer, Guojian Zhang

**Affiliations:** ^1^Department of Chemical and Biological Engineering, University at Buffalo, The State University of New York, Buffalo, NY, United States; ^2^Key Laboratory of Marine Drugs, Chinese Ministry of Education, School of Medicine and Pharmacy, Ocean University of China, Qingdao, China; ^3^Laboratory for Marine Drugs and Bioproducts, Qingdao National Laboratory for Marine Science and Technology, Qingdao, China

**Keywords:** salicylate, salicylate 2-O-β-D-glucoside, metabolic engineering, *E. coli*, analog

## Abstract

Salicylate 2-O-β-D-glucoside (SAG) is a plant-derived natural product with potential utility as both an anti-inflammatory and as a plant protectant compound. Heterologous biosynthesis of SAG has been established in *Escherichia coli* through metabolic engineering of the shikimate pathways and introduction of a heterologous biosynthetic step to allow a more directed route to the salicylate precursor. The final SAG compound resulted from the separate introduction of an *Arabidopsis thaliana* glucosyltransferase enzyme. In this study, a range of heterologous engineering parameters were varied (including biosynthetic pathway construction, expression plasmid, and *E. coli* strain) for the improvement of SAG specific production in conjunction with a system demonstrating improved plasmid stability. In addition, the glucoside moiety of SAG was systematically varied through the introduction of the heterologous oliose and olivose deoxysugar pathways. Production of analogs was observed for each newly constructed pathway, demonstrating biosynthetic diversification potential; however, production titers were reduced relative to the original SAG compound.

## Introduction

Plants have dedicated metabolism for the production of salicylate and a glycosylated version, salicylate 2-O-β-D-glucoside (SAG), which is often stored intracellularly until external stress is encountered (Vlot et al., 2009; Rivas-San Vicente and Plasencia, 2011). At which point, the reversion of SAG to salicylate allows the bioactivity of the latter compound to combat various biological threats to the plant system. Salicylate is also a central component of aspirin and, as such, SAG has the potential to possess similar anti-inflammatory properties.

**FIGURE 1:**
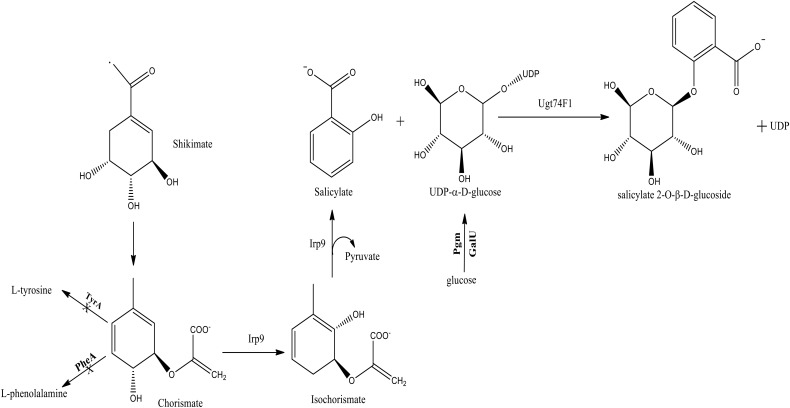
The native and heterologous metabolic pathway for salicylate 2-O-β-D-glucoside (SAG) biosynthesis established within *E. coli*. The native TyrA, PheA, Pgm, and GalU steps were either deleted or over-expressed (bold); whereas the heterologous Irp9 and Ugt74F1 steps were introduced from *Yersinia enterocolitica* and *Arabidopsis thaliana*, respectively.

These various bioactivities of SAG prompted us to explore its production through a heterologous bacterial host. Leveraging the knowledge and prior studies associated with engineering the shikimate pathway of *Escherichia coli* (Lin et al., 2014), we generated a production host supportive of high titer levels of salicylate (>1 g/L) (Ahmadi et al., 2016). This work included the introduction of an Irp9 salicylate synthase gene from *Yersinia enterocolitica*, which streamlined metabolism toward this precursor (**Figure 1**; Pelludat et al., 2003; Kerbarh et al., 2005). The introduction of a glucosyltransferase gene (*ugt74f1*) from *Arabidopsis thaliana* enabled conversion to the final SAG compound (Ahmadi et al., 2016).

In the work presented herein, we were interested in improving SAG production through the application of various heterologous production parameters that spanned the *E. coli* production strain and several expression plasmids and associated components. In addition, based upon previous work by us and others toward glycodiversification of heterologous natural products, we tested the expanded analog potential of the original SAG compound through the systematic incorporation of two isomeric deoxysugar pathways.

## Materials and Methods

### Plasmids and Strains

**FIGURE 2:**
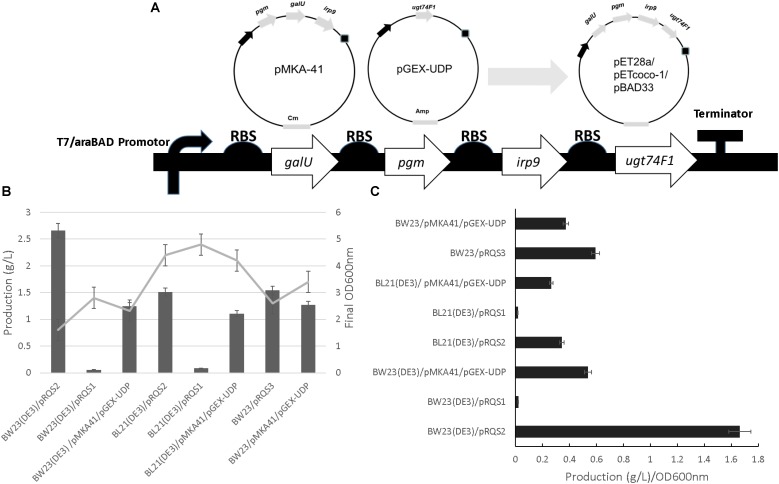
Salicylate 2-O-β-D-glucoside plasmid construction and heterologous production. **(A)** The integration of *irp9, galU, pgm*, and *ugt74F1* into pET28a, pETcoco-1, and pBAD33 expression plasmid backgrounds, yielding pRSQ1, 2, and 3, respectively. Final OD_600nm_ and production levels **(B)** and normalized production per cellular density **(C)** for SAG heterologous production strains.

All cloning procedures were completed in *E. coli* TOP10 through which all recombinant plasmids were transferred and propagated. The *irp9* gene from *Y. enterocolitica* genomic DNA, the *pgm* and *galU* genes from *E. coli* K-12 MG1655, and a codon-optimized *ugt74F1* (salicylate glucosyltransferase gene) from *A. thaliana* were amplified by PCR with primers listed in **Supplementary Table S1**. Plasmids pMKA-41 and pGEX-UDP bearing these genes were constructed as described previously (Ahmadi et al., 2016) and used as the templates for the PCR reactions conducted in the current work. The PCR products were gel-purified and then digested with restriction enzyme pairs *Nhe*I*/Sal*I (for *irp9*), *Xba*I*/Sal*I (for *galU*), and *Nde*I*/Sal*I (for *pgm* and *ugt74F1*). Digested *irp9, pgm, galU*, and *ugt74F1* were separately ligated into similarly digested pET28a to yield pET28-*irp9*, pET28-*pgm*, pET28-*galU*, and pET28a-*ugt74F1*. Plasmid pET28-*pgm* was digested with *Xba*I/*Sal*I and the insert transferred to an *Spe*I/*Sal*I digested pET28-*galU* to yield pET28*-galU*-*pgm*. In the same way, the *Xba*I/*Sal*I digested *ugt74F1* fragment from pET28-*ugt74F1* was inserted into *Spe*I/*Sal*I digested pET28-*irp9* to construct pET28-*irp9*-*ugt74F1*. Plasmid pET28-*irp9*-*ugt74F1* was digested with *Xba*I/*Sal*I and the insert ligated to *Spe*I/*Sal*I digested pET28-*galU*-*pgm* to generate pRQS1. The pRQS1 *galU-pgm-irp9-ugt74F1* cassette featured in **Figure 2** was digested with *Nhe*I*/BsiW*I and ligated into pETcoco-1 for subsequent digestion and transfer of the same cassette (using *Xba*I/*Sal*I) to pBAD33, yielding pRQS2 and pRQS3, respectively. A full list of plasmids and strains are presented in **Supplementary Table S2** and detailed plasmid maps are provided in **Supplementary Figures S1**, **S2**.

The double-knockout BW mutant (BW23) was constructed as described in previous work (Ahmadi et al., 2016) through deletion of *pheA* and *tyrA*, with these deletions improving both SA and SAG production. The λDE3 prophage was integrated into the BW23 chromosome through co-infection using a λDE3 Lysogenization Kit (Novagen), yielding BW23(DE3). By doing so, the new host is equipped with the λDE3 recombinant phage DNA encoding for the T7 RNA polymerase, therefore, allowing the expression of the *galU-pgm-irp9-ugt74F1* cassette though the T7 promotor within the pRQS1 and pRQS2 constructs. The pRSQ plasmids were transformed into corresponding strains through the standard heat-shock transformation protocol (**Supplementary Table S3**), and the resulting strains were stored as 20% glycerol stocks at -80°C.

The gene fragment for *irp9* was liberated from pET28-*irp9* via *Xba*I/*Sal*I digestion and then ligated into *Spe*I/*Sal*I digested pET28-*galU*-*pgm* to construct pET28-*galU*-*pgm*-*irp9*, which was then *Xba*I/*Sal*I digested and ligated into pET21c to generate pRQS4. Plasmids pGJZ1, 2, 3, and 4 (Zhang et al., 2015), containing genes from the oleandomycin, chromomycin, and urdamycin A polyketide biosynthetic pathways, were used to produce two pairs of deoxysugar pathways for oliose and olivose. These four plasmids were integrated with codon-modified *urdGT* to construct pGJZ1-GT, pGJZ2-GT, pGJZ3-GT, and pGJZ4-GT (**Supplementary Figure S3**) which were, respectively, co-transferred with pRQS4 into BL21(DE3) (**Supplementary Figure S4** and **Supplementary Table S4**) to provide the complete biosynthetic pathways for SAG analogs.

### Culture Conditions and Medium Components

The bacterial culture medium and associated chemical and analytical components were obtained from Sigma-Aldrich (St. Louis, MO, United States) or Thermo Fisher Scientific (Waltham, MA, United States). The DNA-manipulating agents, including restriction enzymes, T4 DNA ligase, Phusion High-Fidelity PCR Master Mix, and associated reagents were purchased from New England Biolabs (Ipswich, MA, United States). PCR primers (**Supplementary Table S1**) were obtained from Eurofins Genomics (Huntsville, AL, United States).

Respective glycerol stocks of producing strains from **Supplementary Table S3** were used to initiate overnight 3 mL cultures at 37°C with shaking in lysogeny broth (LB) medium prior to inoculating (1% v/v) 25 mL of M9Y medium which is formulated with (per liter): Na_2_HPO_4_⋅7H_2_O (12.8 g); KH_2_PO_4_ (3 g); NaCl (0.5 g); NH_4_Cl (1 g); yeast extract (1 g), glycerol (10 g), glucose (2.5 g), MgSO_4_⋅7H_2_O (246.5 mg), and CaCl_2_⋅2H_2_O (14.7 mg) supplemented with micronutrients including (per liter) vitamin B1 (2.0 mg), H_3_BO_3_ (1.25 mg), NaMoO_4_⋅2H_2_O (0.15 mg), CoCl_2_⋅6H_2_O (0.7 mg), CuSO_4_⋅5H_2_O (0.25 mg), MnCl2⋅4H_2_O (1.6 mg), and ZnSO_4_⋅7H_2_O (0.3 mg). After inoculation, cultures were incubated at 30°C with shaking for 2 days with induction initiated using 200 μM isopropyl β-D-1-thiogalactopyranoside (IPTG) and/or 3 mg/mL arabinose when the culture OD_600nm_ reached 0.4–0.6. As needed, plasmid selection in both liquid and solid medium was maintained with 100 mg/L ampicillin, 50 mg/L kanamycin, and 20 mg/L chloramphenicol.

### Plasmid Stability Assay

Salicylate 2-O-β-D-glucoside producing strains were cultured in 25 mL production medium containing appropriate antibiotics at 37°C and 250 rpm until the OD_600nm_ reading reached 0.4–0.6. At this point, cultures were cooled to 22°C, induced for gene expression as described above, and incubated an additional 5 days. Plasmid stability analysis was completed on the first and fifth days after cultures were induced. At these time points, dilutions from each culture were spread evenly on LB agar plates for incubation overnight at 37°C. Thirty colonies from each plate were selected and transferred to LB agar plates containing (1) combined antibiotics according to associated gene expression plasmids, (2) 1 mM IPTG, and (3) 1 mM IPTG + combined antibiotics as indicated in **Table 1**. Resulting colony development was then recorded, presented as a percentage of transferred colony growth on LB agar containing no antibiotics, and compared between strains.

**Table 1:** Plasmid stability comparison with antibiotic selection, IPTG induction, and combined IPTG induction and antibiotic selection.

	Antibiotic^∗^		1 mM IPTG^∗∗^		1 mM IPTG + Antibiotic^∗∗∗^	
	Day 1	Day 5	Day 1	Day 5	Day 1	Day 5
BW23(DE3)/pRQS2	100	96.7	0	0	0	3.3
BW23(DE3)/pRQS1	100	100	10	36.7	6.7	66.7
BW23(DE3)/pMKA41/pGEX-UDP	100	83.3	3.3	16.7	0	13.3
BL21(DE3)/pRQS2	100	80	0	0	0	6.7
BL21(DE3)/pRQS1	100	66.7	6.7	26.7	0	56.7
BL21(DE3)/pMKA41/pGEX-UDP	100	53.3	0	6.7	3.3	20
BW23/pRQS3	100	90	0	10	0	10
BW23/pMKA41/pGEX-UDP	96.7	33.3	3.3	13.3	10	23.3

*Numbers represent percentage of transferred colony growth. ^∗^Cells carrying plasmid(s) are able to form colonies on plates containing corresponding antibiotics. ^∗∗^Those strains bearing no plasmid or mutants without the ability to express target genes can grow on plates with 1 mM IPTG. ^∗∗∗^Those strains bearing plasmids but having lost the ability to express the target genes will form colonies on plates with both antibiotics and 1 mM IPTG*.

### Salicylate 2-O-β-D-glucoside (SAG) Production Quantification

Post-culture, 1 mL of acetone was added per 50 mL culture and a 1 mL sample was centrifuged. Supernatant (50 μL) was analyzed by HPLC as described previously (Dean et al., 2005). Briefly, SAG and associated analogs were quantified using a ZORBAX Eclipse XDB-C18 column connected to an Agilent 1100 system equipped with a diode array detector. Solvent A was 0.1% acetic acid in water, solvent B was methanol, and a flow rate of 1 mL/min was used across the following gradient: 5–20% solvent B over 10 min; 20–80% solvent B over 5 min; 80% solvent B maintained for 5 min; reset to 5% solvent B. An absorbance wavelength of 274 nm was used for both SAG and associated analog quantification (Ahmadi et al., 2016). Peak area quantification was conducted compared to a standard calibration curve of pure SAG (Toronto Research Chemicals, Toronto, ON, Canada).

### LC-MS Analysis for SAG Analog Assessment

A 1 mL SAG analog culture sample was centrifuged and 50 μL of supernatant was used for analysis. LC-MS was performed using an API 3000 Triple Quad LC-MS with a Turbo Ion Spray source (PE Sciex) coupled with a Shimadzu Prominence LC system. Chromatography was performed through a Waters XTerra C18 column (5 mm, 2.1 mm × 250 mm) and MS analysis was conducted in positive ion mode. Following a 3 μL injection from the 50 μL sample, a linear gradient of 5–95% acetonitrile (balance water; both solutions containing 0.1% formic acid) was used for 20 min at a flow rate of 0.2 mL/min.

### Statistical Evaluation

Data presented were generated from three independent experiments, and error bars represent standard deviation values.

## Results

As outlined in **Figure 2**, cellular parameters were engineered to improve specific SAG production. First, the production system designed previously for SAG formation [represented by plasmids pMKA-41 and pGEX-UDP (Ahmadi et al., 2016)] was organized into one operon introduced to three different expression plasmids (**Figure 2A**). Plasmids pETcoco-1 and pET28a allowed operon expression from the T7 promoter system coupled to a bacterial strain containing the T7 RNA polymerase (encoded within DE3 cellular variants). The pBAD33 plasmid featured expression driven by an arabinose inducible promoter system (Guzman et al., 1995). Plasmids were then introduced to strains BW23(DE3) and BL21(DE3) to accommodate the T7-based plasmids or BW23 for the pBAD33 plasmid system (also used for pMKA-41 and pGEX-UDP).

A production comparison revealed that the BW23(DE3)/pRSQ2 strain generated the best relative SAG levels based upon volumetric and specific titer comparisons (**Figures 2B,C** and **Supplementary Figure S5**). When comparing performance by either SAG titer or production per cell density, the BW23(DE3)/pRSQ2 strain demonstrated a twofold to fivefold improvement relative to the original BW23/pMKA-41/pGEX-UDP system. Of the new expression plasmids tested, pRSQ1 showed the lowest levels of SAG production. Of the production strains tested, the BW23(DE3) background, engineered to support SAG metabolic channeling and to accommodate the strong T7 promoter, demonstrated the best overall titers.

The plasmids used in this study included a low-copy option (pRSQ2; pETcoco-1 [OriV/S, 1–2 copies per cell]) and two medium copy options: pRSQ1 (pET28a [pBR322, ∼40 copies per cell]) and pRSQ3 (pBAD33 [pACYC184/p15A]). **Table 1** presents plasmid stability data for the associated SAG production strains. From this analysis, the RSQ2 plasmid shows the best overall stability when tested for plasmid maintenance over time. The consolidated SAG biosynthetic pathways across plasmids pRSQ1-3 showed improved stability relative to the original dual expression plasmid system reliant upon pMKA-41 and pGEX-UDP. The same set of strains were also tested for stability when exposed to either IPTG induction or IPTG induction with antibiotic selection Novagen (1999). The plasmid stability data under these conditions indicate that, with the exception of pRSQ2, the newly constructed systems suffer from a combination of plasmid loss and mutant formation that results in lack of gene expression. From this perspective, the order of plasmid stability would be pRSQ2, pRSQ3, and pRSQ1.

**FIGURE 3:**
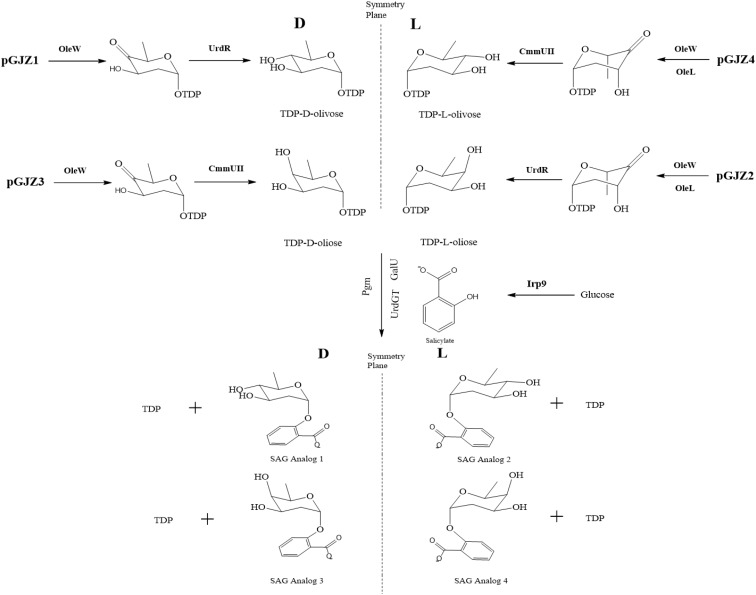
Biosynthetic pathways for the production of SAG analogs. pGJZ plasmids 1–4 encode pairs of chiral-symmetric olivose and oliose deoxysugars to glycodiversify salicylate.

**FIGURE 4:**
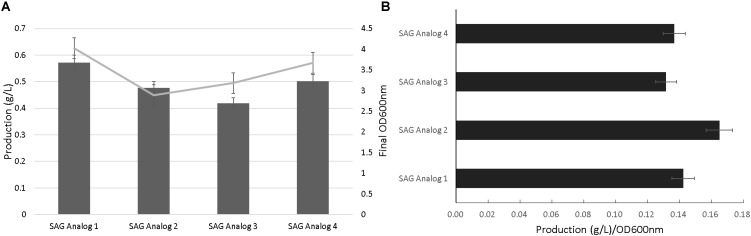
Salicylate 2-O-β-D-glucoside analog production. Final OD_600nm_ and production levels **(A)** and normalized production per cellular density **(B)**.

**Figure 3** outlines a schematic to test the flexibility of the SAG pathway to generate analogs resulting from alternative glycosylation patterning. In particular, isomeric variants of the oliose and olivose deoxysugars were tested for glycodiversification of the incoming salicylate precursor (Aguirrezabalaga et al., 2000). Initial analog production efforts with the Ugt74F1 salicylate glucosyltransferase resulted in minimal product formation (data not shown). As an alternative, the urdamycin system glycotransferase (UrdGT) was used due to previously observed flexibility in glycosylation patterning (Hoffmeister et al., 2000, 2003). Using HPLC and LC-MS analysis, data supporting analog formation were generated for each deoxysugar variant (**Figures 4A,B** and **Supplementary Figures S6**, **S7**). However, production levels were significantly reduced compared to those from the original SAG production systems (**Figure 2**).

## Discussion

A combination of metabolic engineering and gene expression design resulted in plasmids pRSQ1, 2, and 3. Each plasmid design consolidated genes needed for SAG biosynthesis. Furthermore, the plasmids featured different copy numbers and promoter strengths and, when combined with *E. coli* strains engineered to support gene expression and SAG production, allowed for a systematic evaluation of final product values. Production levels were best for strain BW23(DE3)/pRSQ2, which featured a metabolically engineered strain to streamline carbon flow to SAG production and support the strong T7 promoter driving expression from pRSQ2. Of note, this particular expression plasmid was the lowest copy version of those tested. pRSQ1 featured T7-based gene expression but from a higher copy plasmid; pRSQ3 utilized an arabinose-inducible system within pBAD33 (similar to use previously with the control system BW23/pMDA-41/pGEX-UDP). This particular study focused only on SAG production as a function of plasmid biosynthetic pathway design (**Figure 2**); however, future engineering approaches, such as tuning biosynthetic steps via expression variation, will likely be needed to maximally drive complete salicylate to SAG formation (Ahmadi et al., 2016).

One likely contributor to the heightened production observed for pRSQ2 was incorporation of the highly stable OriV/S replication system (Shizuya et al., 1992; Monaco and Larin, 1994; Tao and Zhang, 1998). As such, even though the copy number for this plasmid was reduced, stability was improved as indicated within the results presented in **Table 1**. The strong T7 expression system likely compensated for the reduced copy number level (Golomb and Chamberlin, 1974; Studier and Moffatt, 1986); whereas, the higher copy number T7 system represented by pRSQ1 showed lower relative SAG production and higher plasmid loss. The pRSQ2 system has the added advantage (not tested in this study) of plasmid copy-up capability (Wild et al., 1996, 2001, 2002; Wild and Szybalski, 2004). Thus, there is the potential for further increased production through the stable maintenance of the pRSQ2 plasmid during the growth phase of the host system followed by induction of both SAG biosynthesis and plasmid amplification to spur subsequent product generation.

Our group and others have studied natural product glycodiversification, which offers a directed means of natural product structural variation (Thibodeaux et al., 2007, 2008; Williams et al., 2008; Jiang et al., 2013; Zhang et al., 2015; Fang et al., 2018). Given the glycosylated nature of SAG, we were interested if this product could also accommodate alternative sugar moieties. In conducting this work, we relied on a series of deoxysugar pathways previously incorporated into polyketide biosynthesis to generate erythromycin analogs (Zhang et al., 2015). However, contingent upon this strategy working is the flexibility of a glycosylation enzymatic step capable of accepting new substrate groups. Use of the original glucosyltransferas (Ugt74F1) resulted in minimal analog production. As a result, we turned to an alternative glycotransferase from the urdamycin biosynthetic pathway [recognized for substrate flexibility (Hoffmeister et al., 2002; Luzhetskyy et al., 2005) and the same source as some of the deoxysugar pathways genes], which resulted in the production level of the analogs presented in **Figure 4**. The ability for the urdamycin glycotransferase to accommodate novel SAG analogs supports the general theme of glycodiversification applied to this compound. As in the case of several previous examples of analogs produced as a result of biosynthetic pathway modification (Jiang and Pfeifer, 2013; Zhang et al., 2015), titer levels of the SAG analogs were significantly reduced compared to the original product, likely to do the new substrates limiting the catalytic activity of the glycosyltransferase. We also note that additional analytical work is needed to fully chemically characterize these new analogs. However, indication of novel analogs provides a basis for future studies to test potential variation in bioactivity in applications that range from inflammatory relief [as we have tested previously with the original SAG compound (Ahmadi et al., 2016)] or plant stress protection.

## Author Contributions

GZ and BP designed and supervised the study. RQ executed the experimental plan.

## Conflict of Interest Statement

The authors declare that the research was conducted in the absence of any commercial or financial relationships that could be construed as a potential conflict of interest.
