# Supplementary material for: Engineering Heterologous Production of Salicylate Glucoside and Glycosylated Variants
Source: Front Microbiol. 2018 Sep 20;9:2241. doi: 10.3389/fmicb.2018.02241 (PMC6158457; doi:10.3389/fmicb.2018.02241)
Supplement: Supplementary file 1 [file Data_Sheet_1.pdf]

Supplementary Material

Engineering Heterologous Production of Salicylate Glucoside and Glycosylated Variants

Ruiquan Qi[1], Blaine A. Pfeifer[1,2,3*], Guojian Zhang[2,3*]

[1]Department of Chemical and Biological Engineering, University at Buffalo, The State University of New York, Buffalo, New York, 14260, USA

[2]Key Laboratory of Marine Drugs, Chinese Ministry of Education, School of Medicine and Pharmacy, Ocean University of China, Qingdao 266003, People's Republic of China

[3]Laboratory for Marine Drugs and Bioproducts of Qingdao National Laboratory for Marine Science and Technology, Qingdao, 266237, People's Republic of China

***Correspondence:**

Blaine A. Pfeifer

blainepf@buffalo.edu

Guojian Zhang

guojianz@buffalo.edu

Supplementary Tables

Table S1. PCR primers used in this study. Restriction sequences are bolded.

Primer	Sequence
irp9-F	5'-CTA**GCTAGC**ATGAAATCAGTGAATTTCT-3'
irp9-R	5'-ACGC**GTCGACACTAGT**CTACTACACCATTAAATAGGG-3'
galU-F	5'-GC**TCTAGA**AATAATTTTGTTTAACTTTAAGAAGGAGATATA ATGGCTGCCATTAATACGAAA-3'
galU-R	5'-ACGC**GTCGACACTAGT**TTACTTCTTAATGCCCATCTC-3'
pgm-F	5'-GGAATTCC**CATATG**GCAATCCACAATCGTGCA-3'
pgm-R	5'-ACGC**GTCGACACTAGT**TTACGCGTTTTTCAGAACTTC-3'
ugt74F1-F	5'-GAATTC**CATATG**GAGAAGATGCGTGG-3'
ugt74F1-R	5'-ACGC**GTCGACCGTACGACTAGT**TTTGATCTGGATCTTGCA-3'

Table S2. Plasmids and strains used in this study

**Plasmid**	**Description**	**Reference/Source**
pET28a	pT7, pBR322 ori, Kan^R	Novagen
pET21c	pT7, pBR322 ori, Amp^R	Novagen
pBAD33	pBAD, pACYC184/p15A ori, Cm^R	[1]
pETcoco-1	pT7, OriV/S, Cm^R	Novagen
pET28-*irp9*	pET28a harboring *irp9* from *Yersinia enterocolitica*	This study
pET28-*pgm*	pET28a harboring *pgm* from *E. coli* K-12 MG1655	This study
pET28-*galU*	pET28a harboring *galU* from *E. coli* K-12 MG1655	This study
pET28-*ugt74F1*	pET28a harboring UDP-glycosyltransferase (*ugt74F1*) from *Arabidopsis thaliana*	This study
pET28-*galU-pgm*	pET28a harboring *galU* and *pgm* in an operon configuration	This study
pET28-*irp9-ugt74F1*	pET28a harboring *irp9* and *ugt74F1* in an operon configuration	This study
pET28-*galU-pgm-irp9*	pET28a harboring *galU, pgm,* and *irp9* in an operon configuration	This study
pRQS1	pET28a harboring *galU*, *pgm*, *irp9*, and *ugt74F1* in an operon configuration	This study
pRQS2	pETcoco-1 harboring *galU*, *pgm*, *irp9*, and *ugt74F1* in an operon configuration	This study
pRQS3	pBAD harboring *galU*, *pgm*, *irp9*, and *ugt74F1* in an operon configuration	This study
pRQS4	pET21c harboring *galU*, *pgm*, and *irp9* in an operon configuration	This study
pMKA-41	pBAD harboring *irp9*, *galU*, and *pgm* in an operon configuration, Cm^R	[2]
pGEX-UDP	pGEX-2TK harboring *ugt74F1* from *Arabidopsis thaliana*, Amp^R	[2]
pGJZ1	pET28a harboring *oleV*, *oleW*, and *urdR*, Kan^R	[3]
pGJZ2	pET28a harboring *oleV*, *oleW*, *oleL*, and *urdR*, Kan^R	[3]
pGJZ3	pET28a harboring *oleV*, *oleW*, and *cmmUII*, Kan^R	[3]
pGJZ4	pET28a harboring *oleV*, *oleW*, *oleL*, and *cmmUII*, Kan^R	[3]
pGJZ1-GT	pGJZ1 integrated with codon-modified UrdGT gene (*urdGTm*)	This study
pGJZ2-GT	pGJZ2 integrated with codon-modified *urdGTm*	This study
pGJZ3-GT	pGJZ3 integrated with codon-modified *urdGTm*	This study
pGJZ4-GT	pGJZ4 integrated with codon-modified *urdGTm*	This study
**Strain**	**Genotype**	**Source**
BL21(DE3)	F- *ompT hsdSB* (rB-mB-) *gal dcm* (DE3)	Novagen
BW25113	F-, Δ(*araD-araB*)567, Δ*lacZ*4787(::*rrnB*-3), LAM-, rph-1, Δ(*rhaD-rhaB*)568, *hsdR*514	[4]
BW23	BW25113::Δ*pheA*, Δ*tyrA*	[2]
BW23(DE3)	BW25113::Δ*pheA*, Δ*tyrA* equipped with λDE3	This study

Table S3. SAG producing strains developed in this study.

Host	Plasmid transformed	Control	Inducer
BL21(DE3)	pRQS1	pET28a	IPTG
BL21(DE3)	pRQS2	pETcoco-1	IPTG
BL21(DE3)	pMK41, pGEX-UDP	pBAD33/pGEX-2TK	Arbinose/IPTG
BW23(DE3)	pRQS1	pET28a	IPTG
BW23(DE3)	pRQS2	pETcoco-1	IPTG
BW23(DE3)	pMK41, pGEX-UDP	pBAD33/pGEX-2TK	Arbinose/IPTG
BW23	pRQS3	pBAD33	Arbinose
BW23	pMK41, pGEX-UDP	pBAD33/pGEX-2TK	Arbinose/IPTG

Table S4. SAG analog producing strains developed in this study.

Host	Plasmid transformed	Control	Inducer
BL21(DE3)	pRQS4+pGJZ1-GT	pET21c+pET28a	IPTG
BL21(DE3)	pRQS4+pGJZ2-GT		IPTG
BL21(DE3)	pRQS4+pGJZ3-GT		IPTG
BL21(DE3)	pRQS4+pGJZ4-GT		IPTG

Supplementary Figures

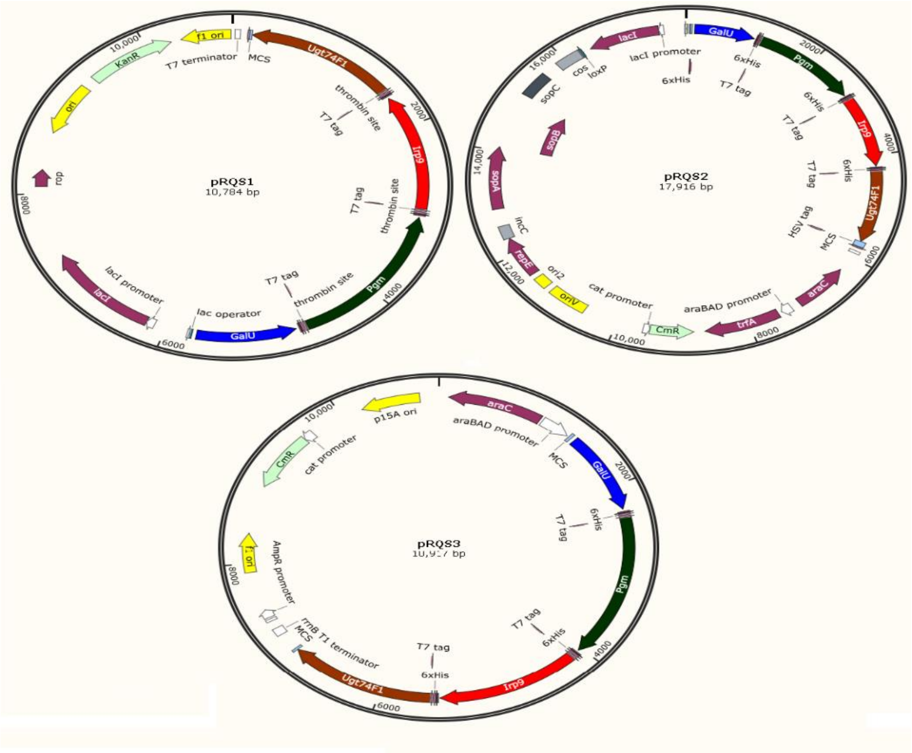

Figure S1. Maps of pRQS plasmids used to produce SAG.

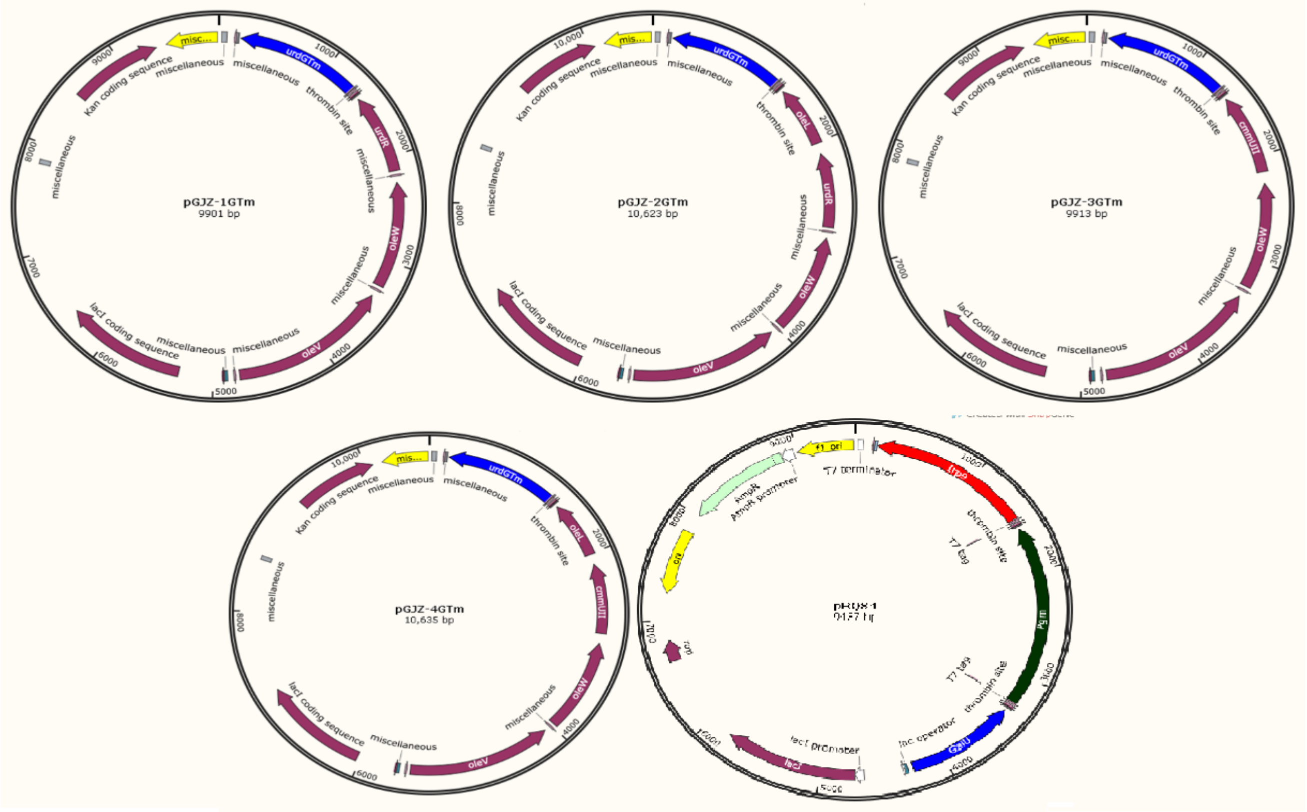

Figure S2. Maps of plasmids used to produce SAG analogs.

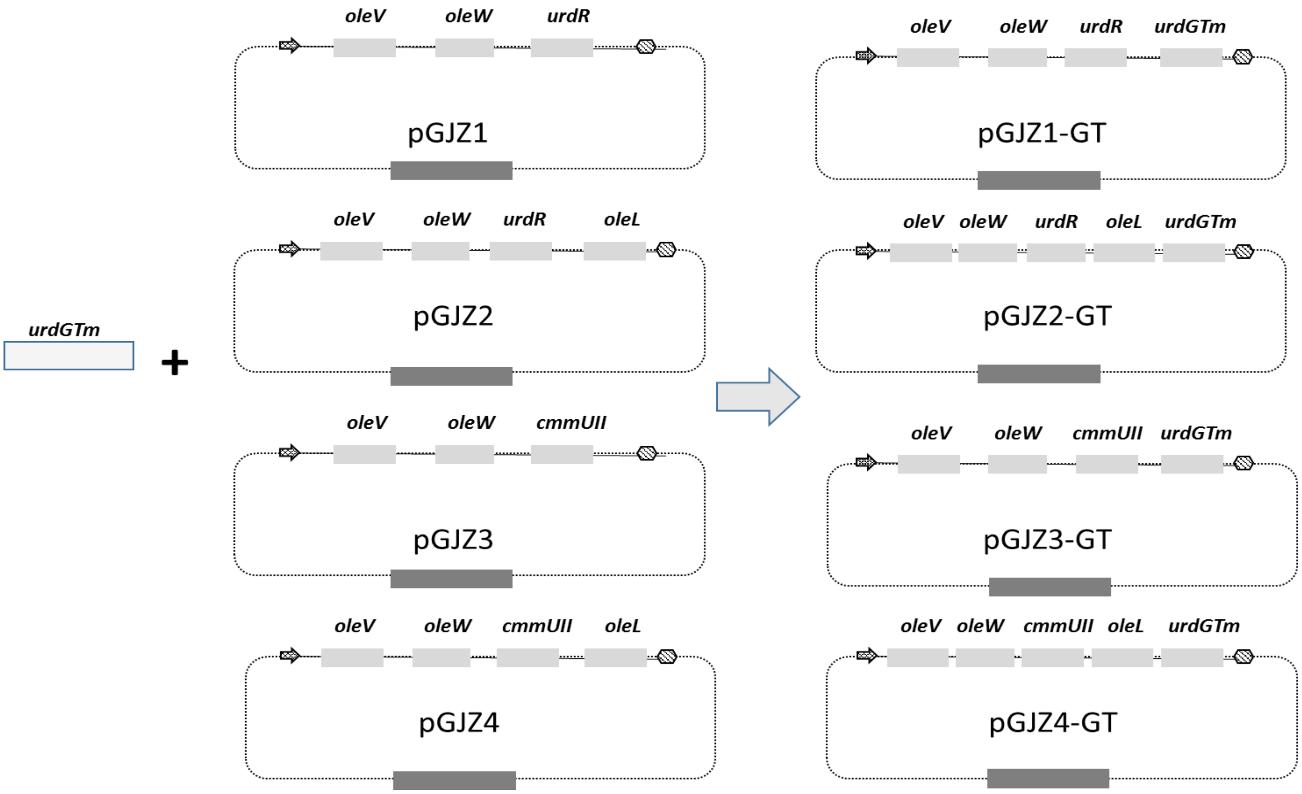

Figure S3 Construction of deoxysugar producing plasmids. The codon-modified *urdGTm* gene was integrated into pGJZ1, 2, 3 and 4 to produce isomeric variants of oliose and olivose shown in Fig. 3.

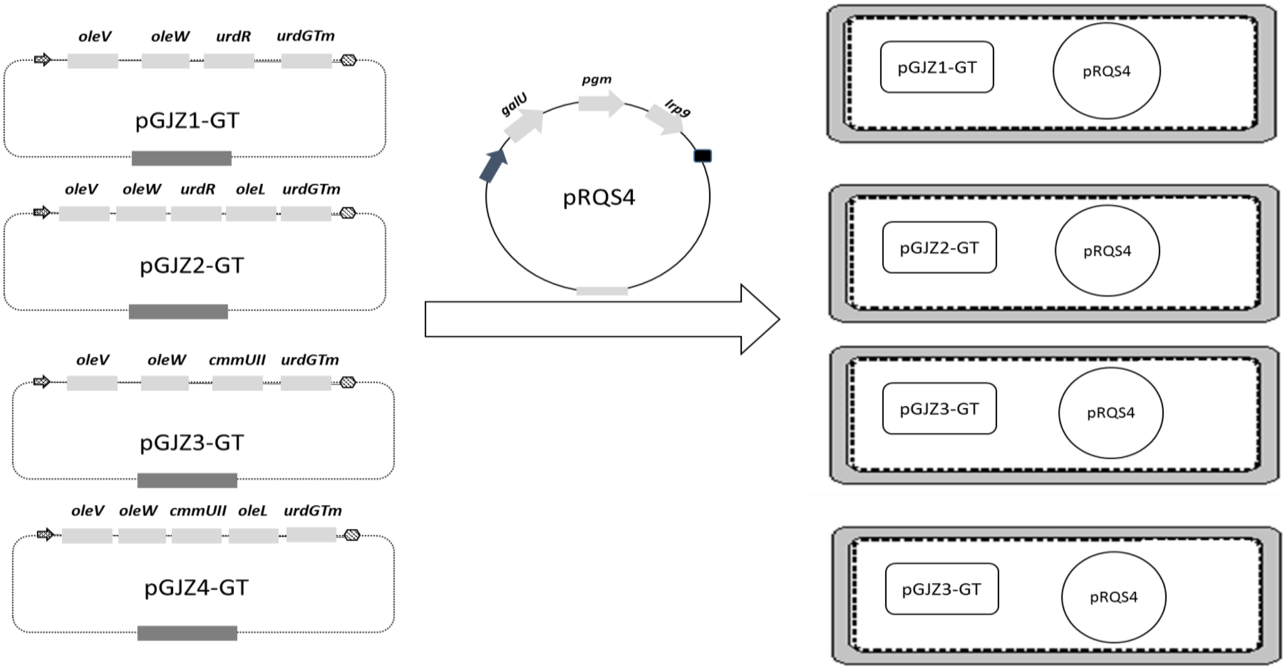

Figure S4 SAG analog producing strain development in which four recombinant plasmids (producing oliose and olivose chiral pairs) are co-transformed with pRQS4 into BL21(DE3).

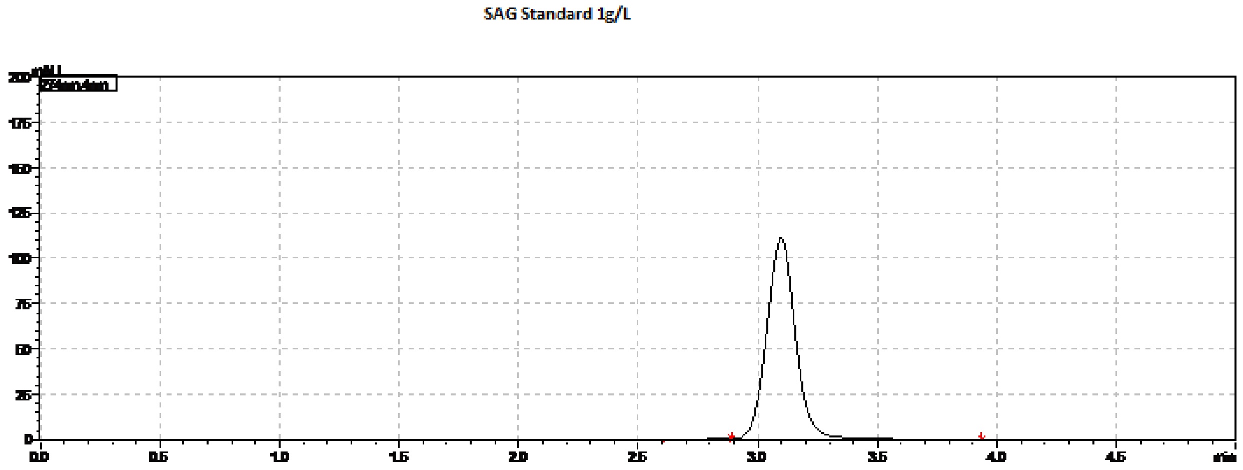

Figure S5a. HPLC trace of authentic SAG standard (1 g/L).

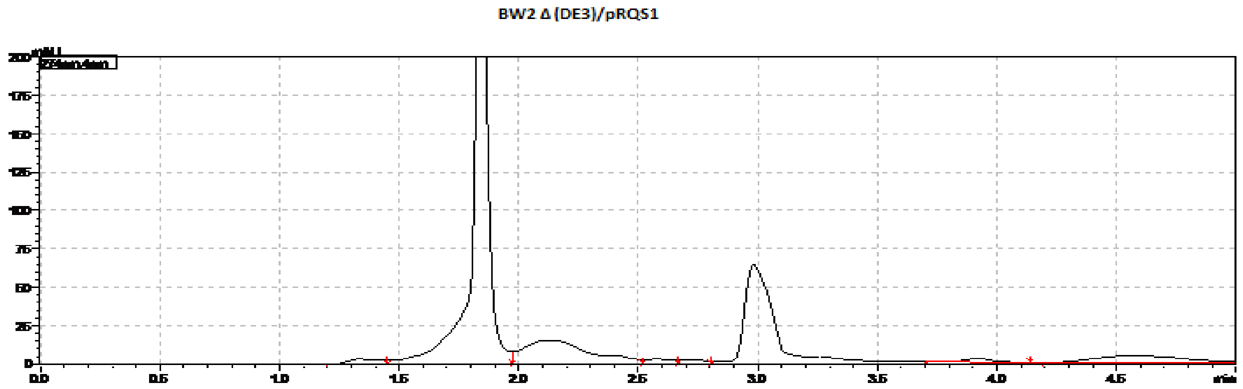

Figure S5b. HPLC trace of SAG heterologous production in strain BW23(DE3)/pRQS1.

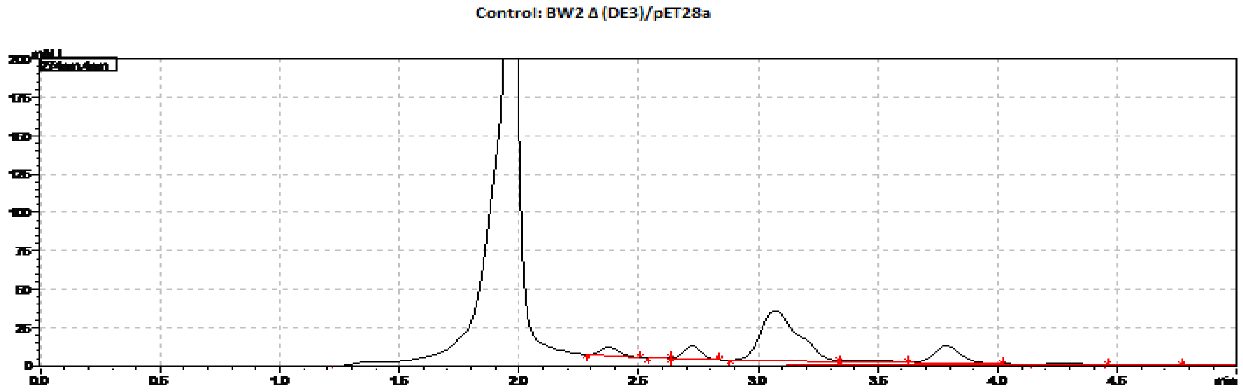

Figure S5c. HPLC trace of control strain BW23(DE3)/pET28a.

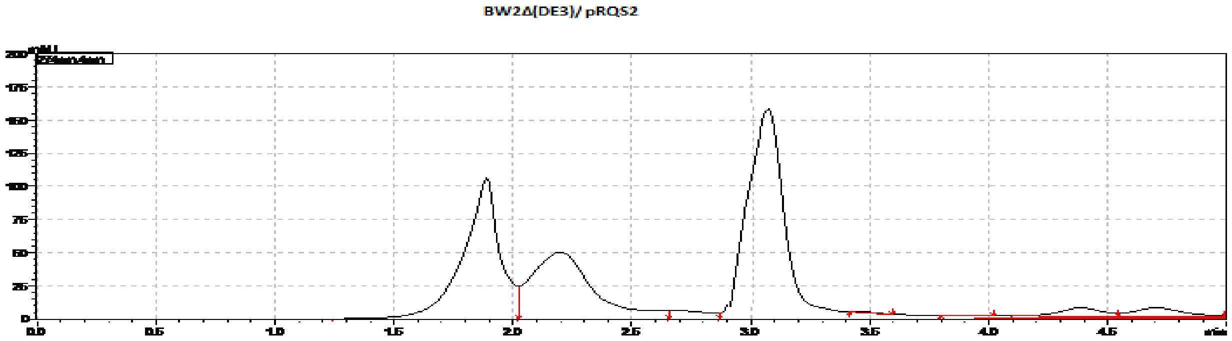

Figure S5d. HPLC trace of SAG heterologous production in strain BW23(DE3)/pRQS2.

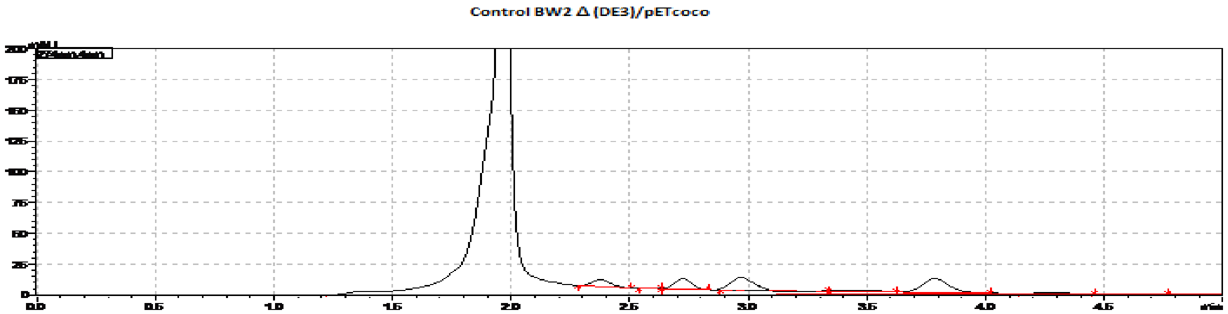

Figure S5e. HPLC trace of control strain BW23(DE3)/pETcoco-1.

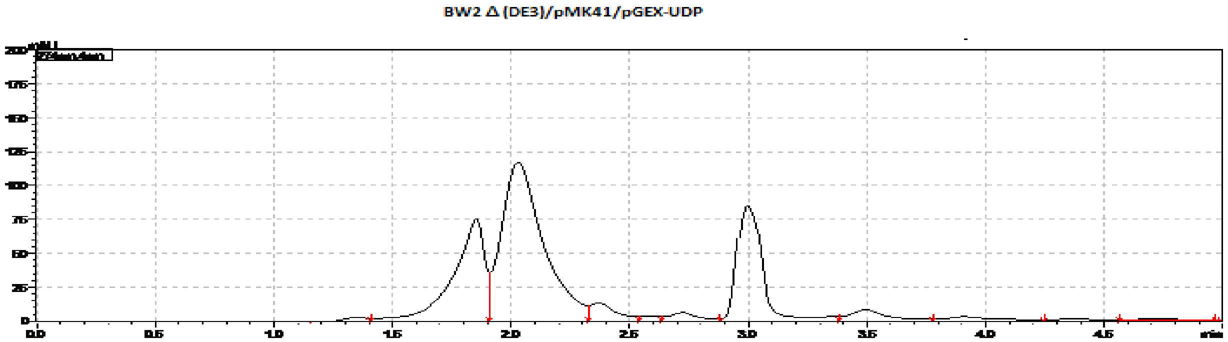

Figure S5f. HPLC trace of SAG heterologous production in strain BW23(DE3)/pMKA-41/pGEX-UDP.

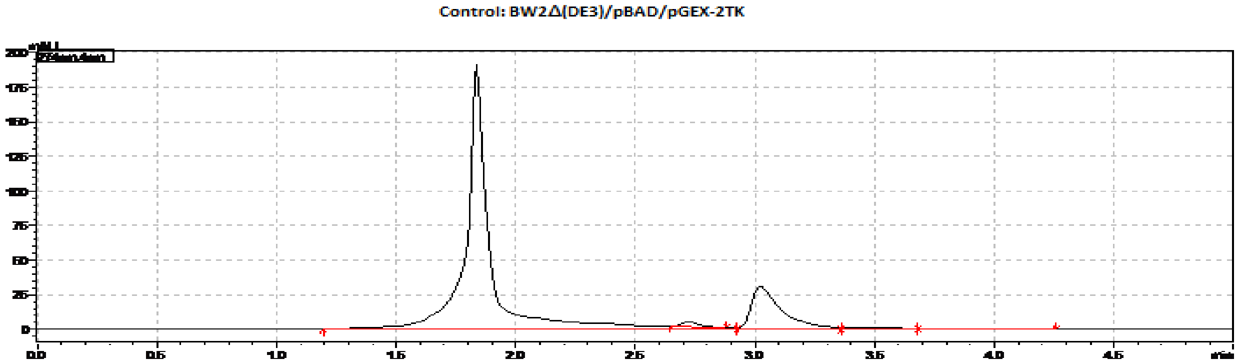

Figure S5g. HPLC trace of control strain BW23(DE3)/pBAD33/pGEX-2TK.

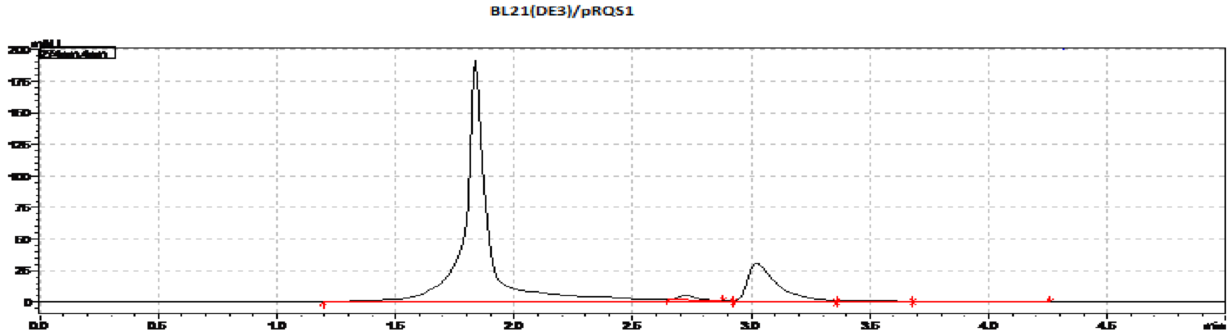

Figure S5h. HPLC trace of SAG heterologous production in strain BL21(DE3)/pRQS1.

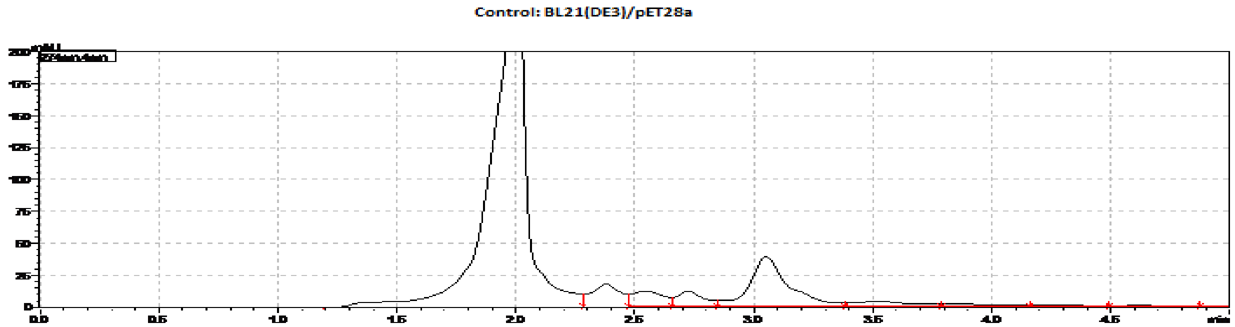

Figure S5i. HPLC trace of control strain BL21(DE3)/pET28a.

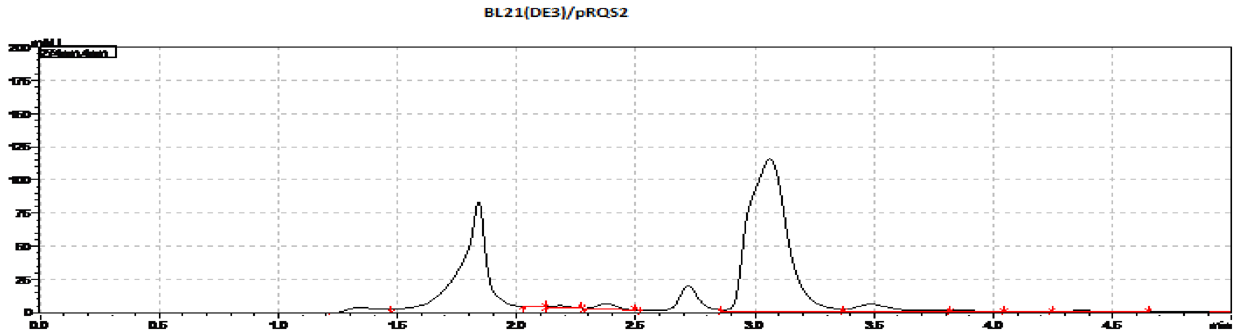

Figure S5j. HPLC trace of SAG heterologous production in strain BL21(DE3)/pRQS2.

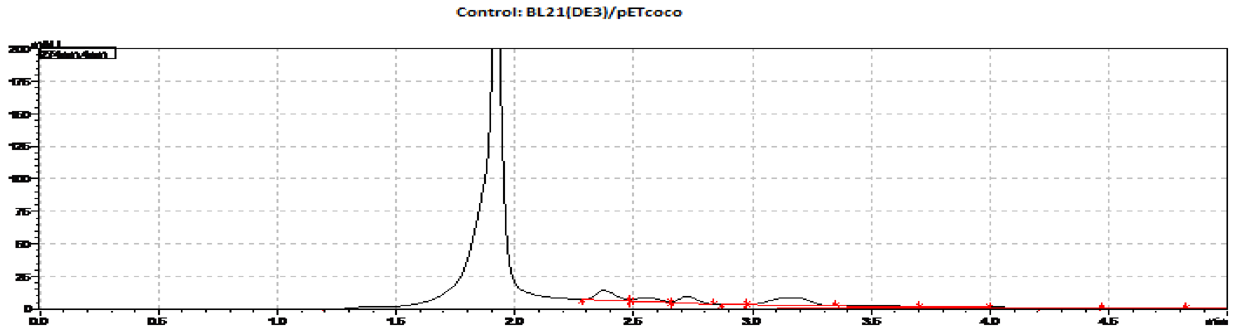

Figure S5k. HPLC trace of control strain BL21(DE3)/pETcoco-1.

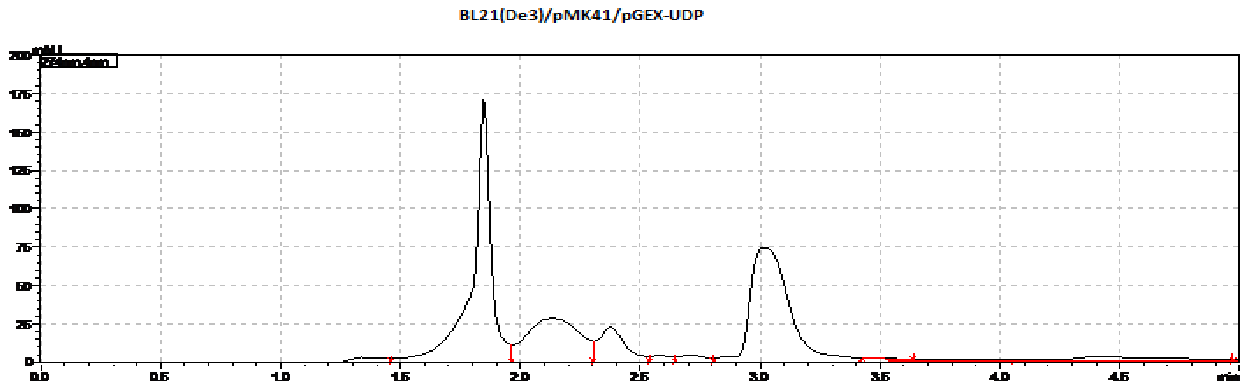

Figure S5l. HPLC trace of SAG heterologous production in strain BL21(DE3)/pMKA-41/pGEX-UDP.

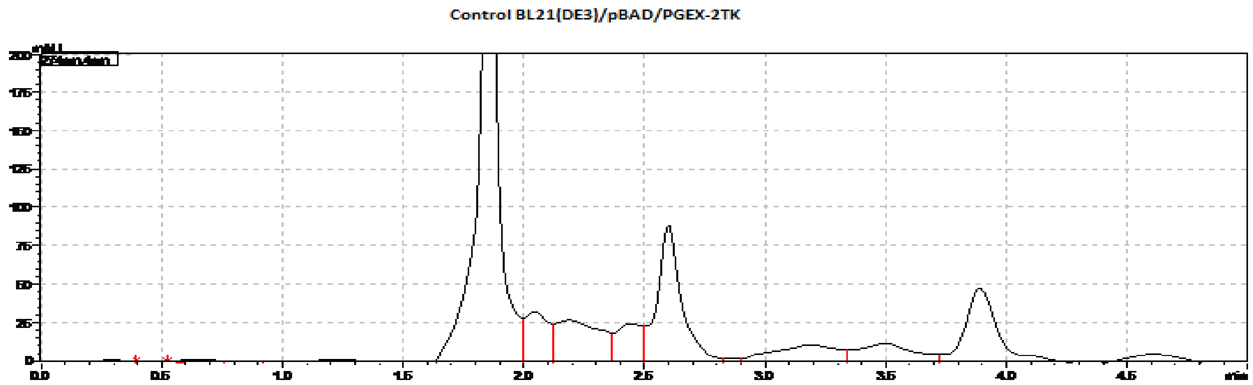

Figure S5m. HPLC trace of control strain BL21(DE3)/pBAD33/pGEX-2TK.

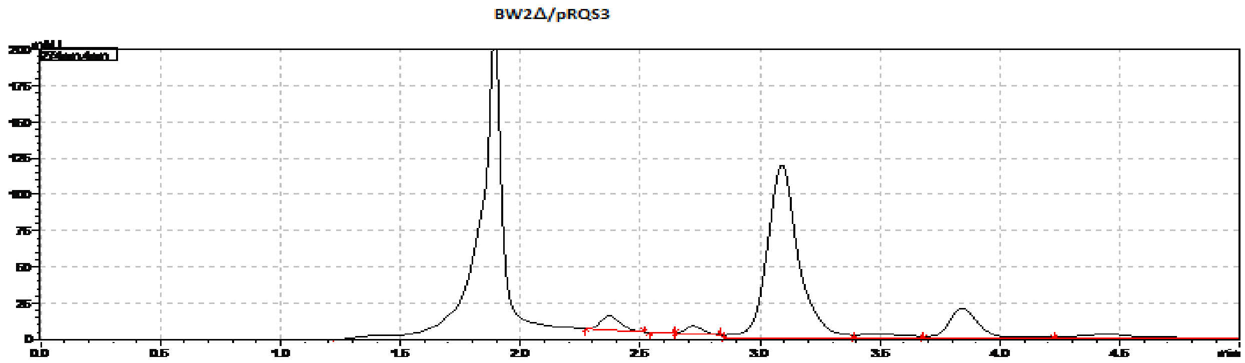

Figure S5n. HPLC trace of SAG heterologous production in strain BW23/pRQ3.

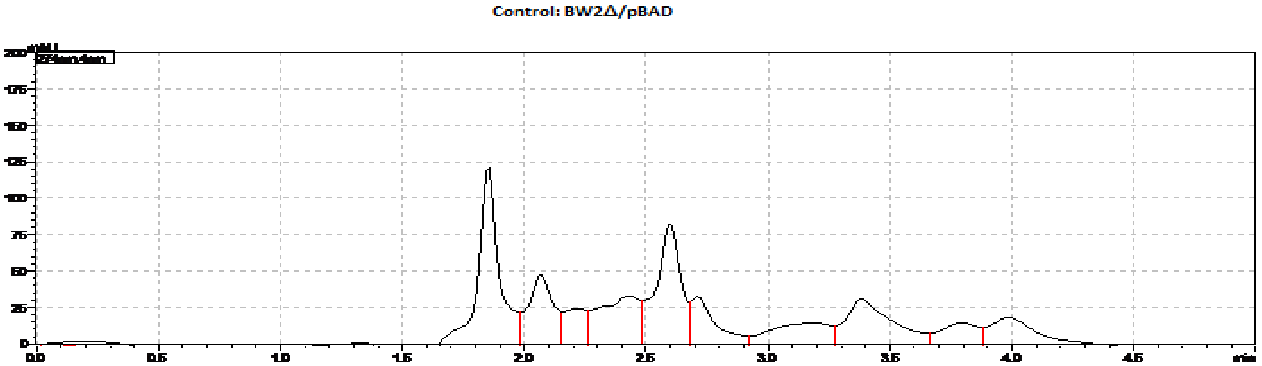

Figure S5o. HPLC trace of control strain BW23/pBAD33.

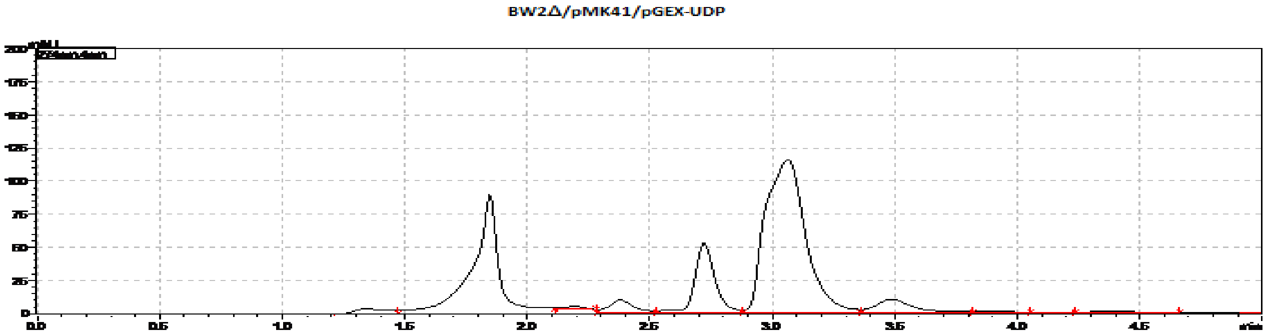

Figure S5p. HPLC trace of SAG heterologous production in strain BW23/pMKA-41/pGEX-UDP.

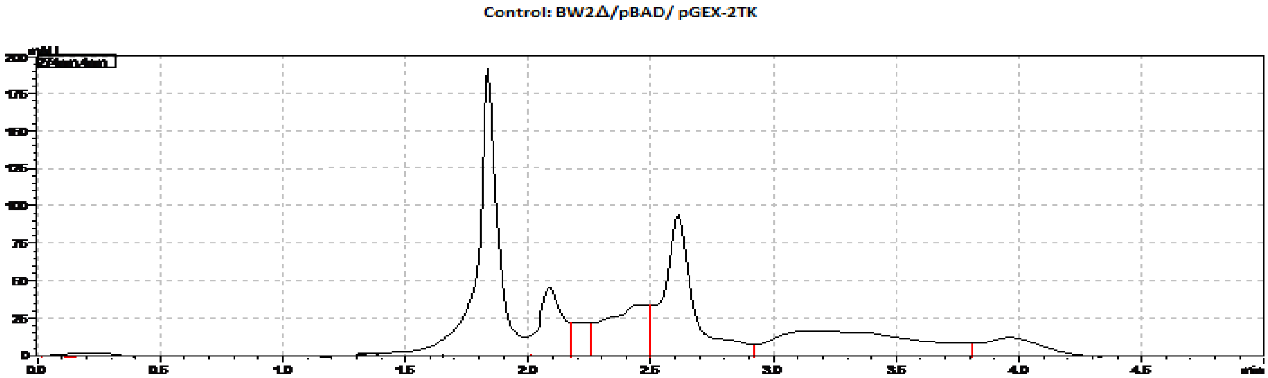

Figure S5q. HPLC trace of control strain BW23/pBAD33/pGEX-2TK

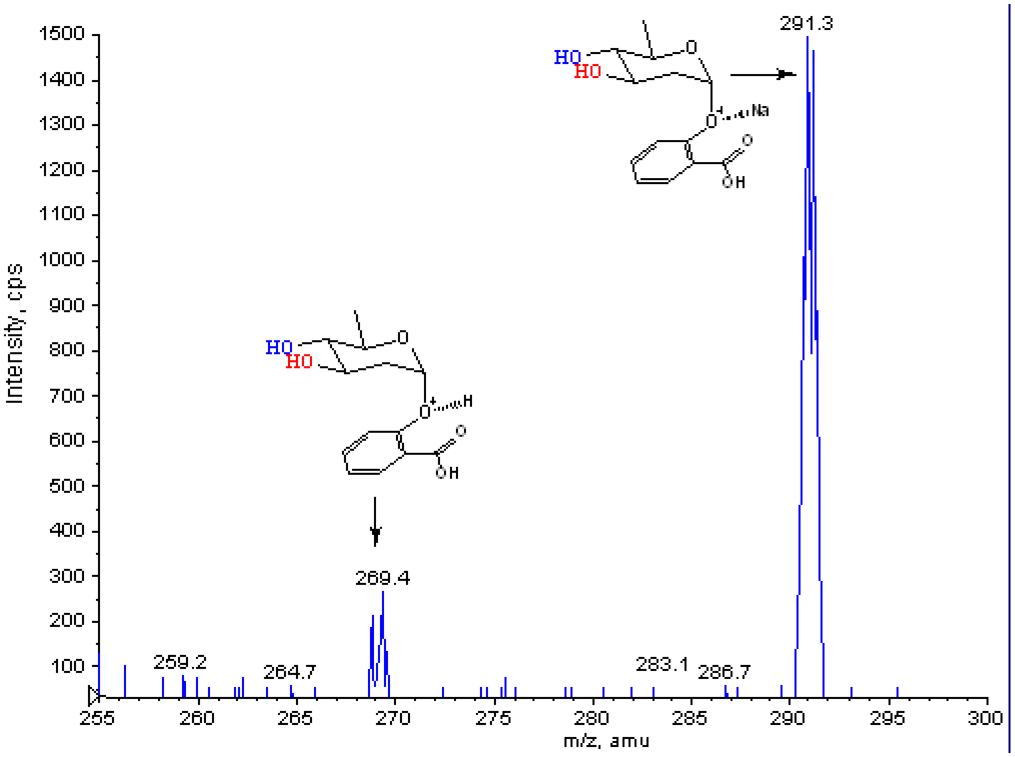

Figure S6a. LC-MS spectrum for SAG analog 1. m/z 269.4 $[M + H]^+$ and 291.3 $[M + Na]^+$ (calcd. for $C_{13}H_{16}O_6$, 268.3).

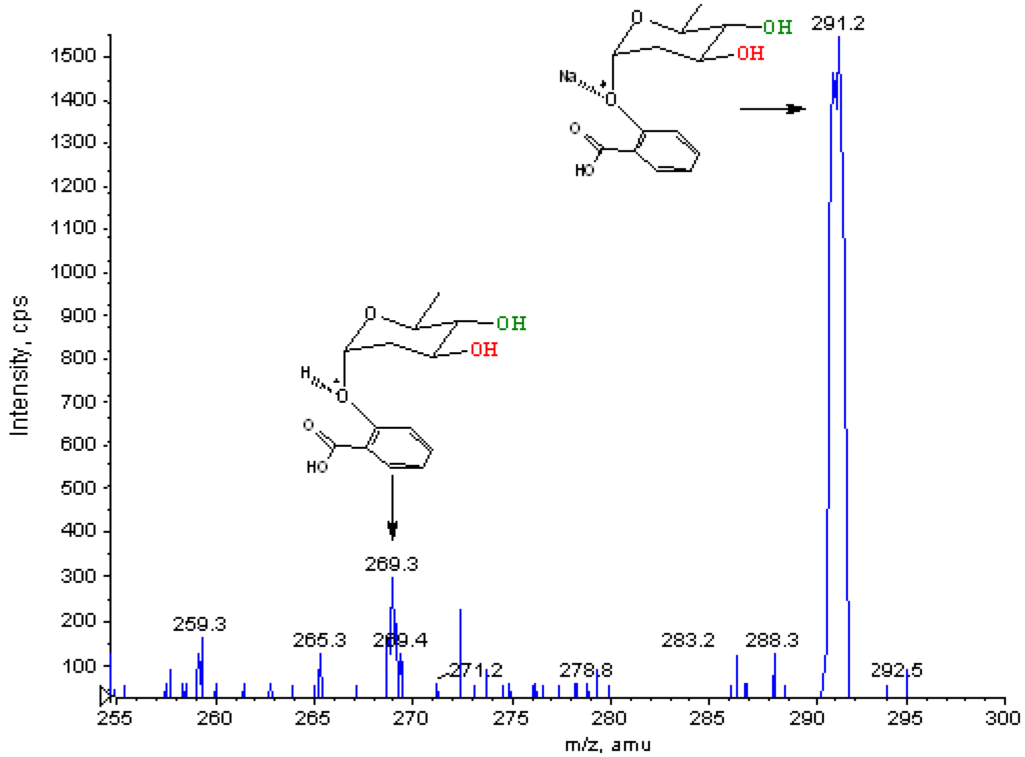

Figure S6b. LC-MS spectrum for SAG analog 2. m/z 269.3 $[M + H]^+$ and 291.2 $[M + Na]^+$ (calcd. for $C_{13}H_{16}O_6$, 268.3).

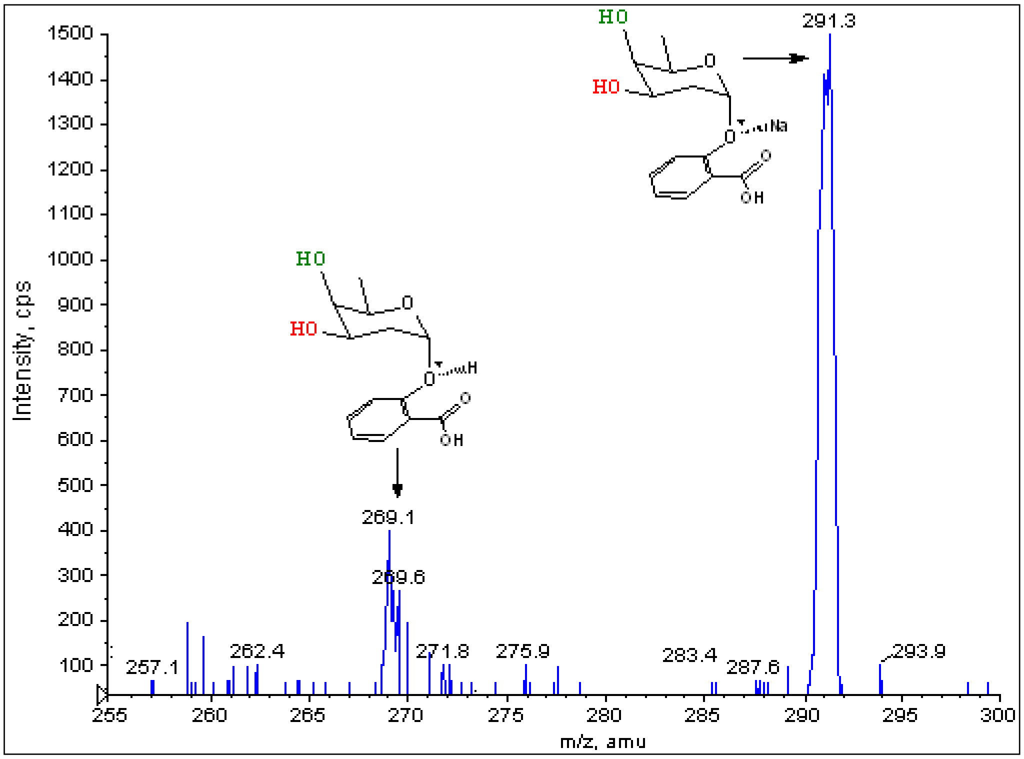

Figure S6c. LC-MS spectrum for SAG analog 3. m/z 269.1 $[M + H]^+$ and 291.3 $[M + Na]^+$ (calcd. for $C_{13}H_{16}O_6$, 268.3).

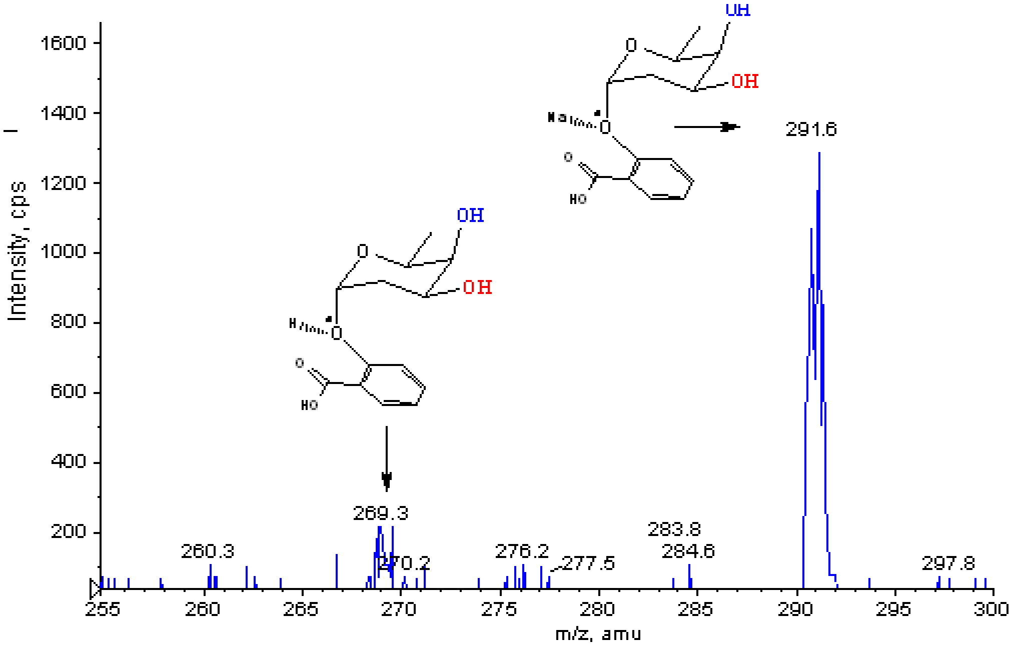

Figure S6d. LC-MS spectrum for SAG analog 4. m/z 269.3 $[M + H]^+$ and 291.6 $[M + Na]^+$ (calcd. for $C_{13}H_{16}O_6$, 268.3).

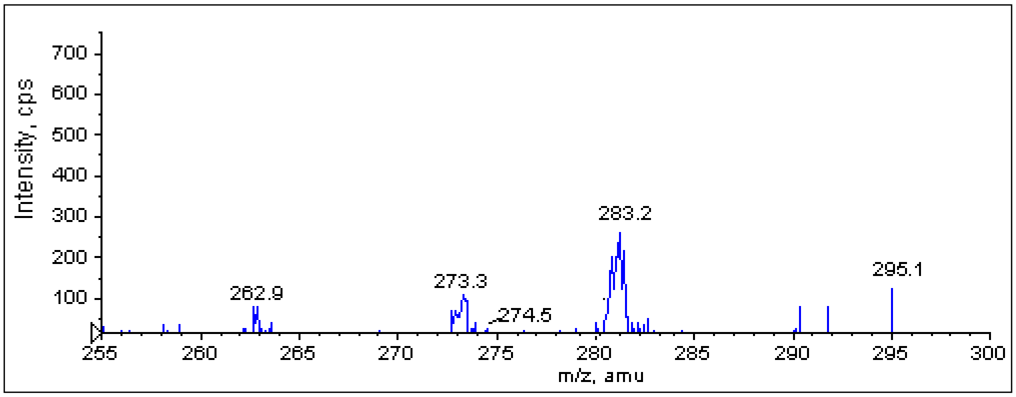

Figure S6e. LC-MS spectrum of SAG analog control strain.

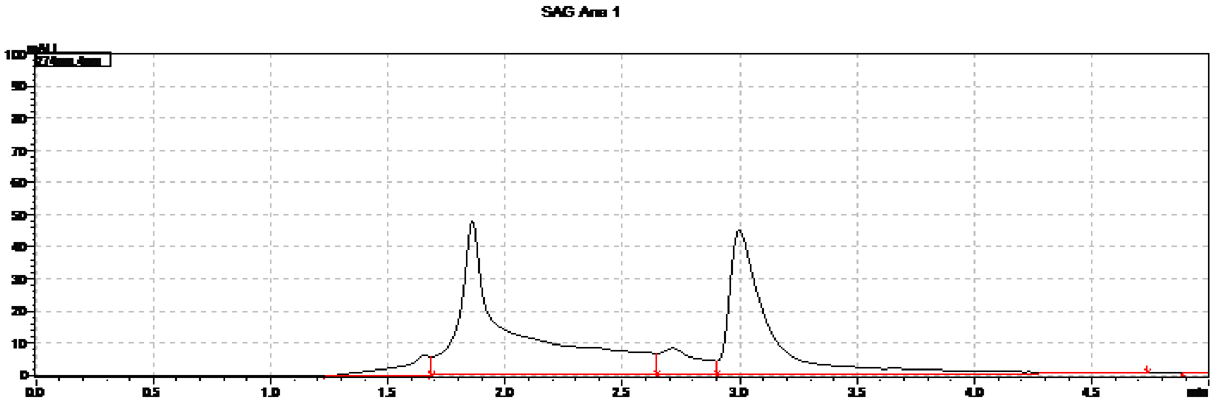

Figure S7a. HPLC trace of SAG Analog 1 heterologous production in strain BL21(DE3)/pGJZ1-GT/pRQS4.

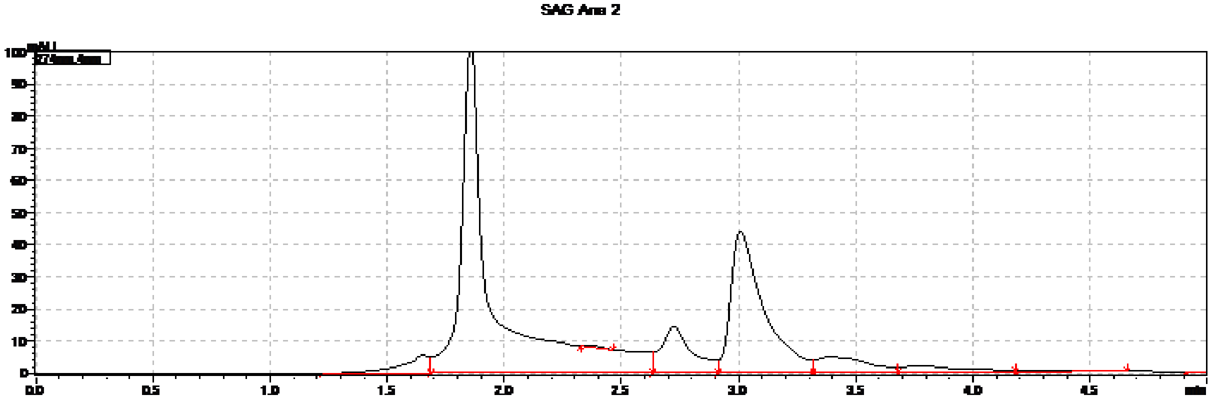

Figure S7b. HPLC trace of SAG Analog 2 heterologous production in strain BL21(DE3)/pGJZ2-GT/pRQS4.

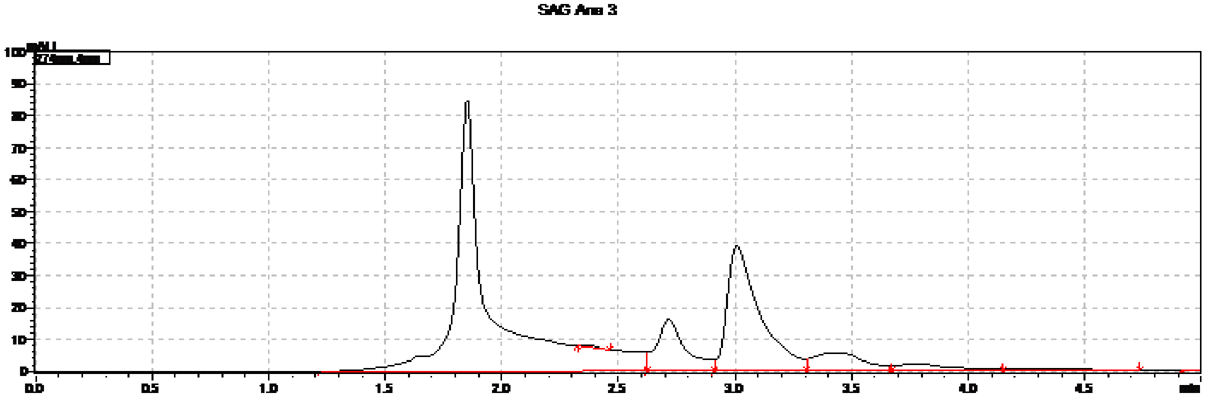

Figure S7c. HPLC trace of SAG Analog 3 heterologous production in strain BL21(DE3)/pGJZ3-GT/pRQS4.

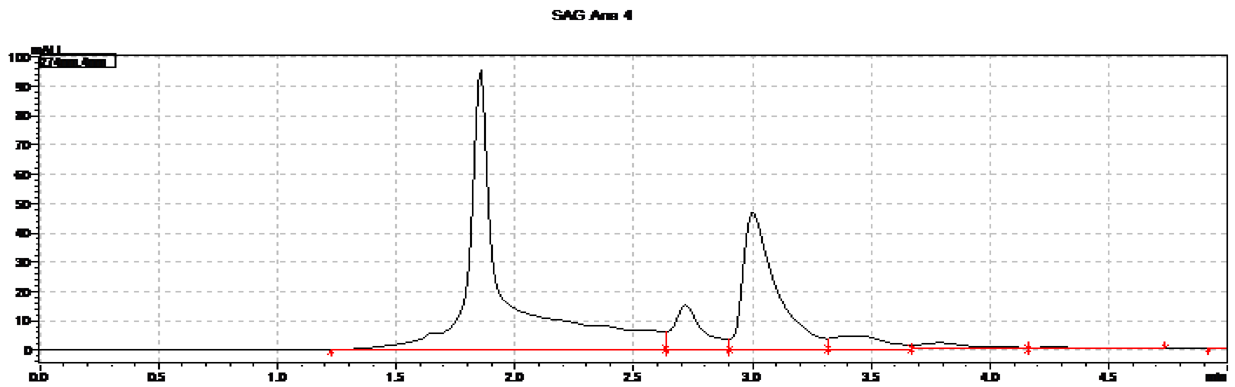

Figure S7d. HPLC trace of SAG Analog 4 heterologous production in strain BL21(DE3)/pGJZ4-GT/pRQS4.

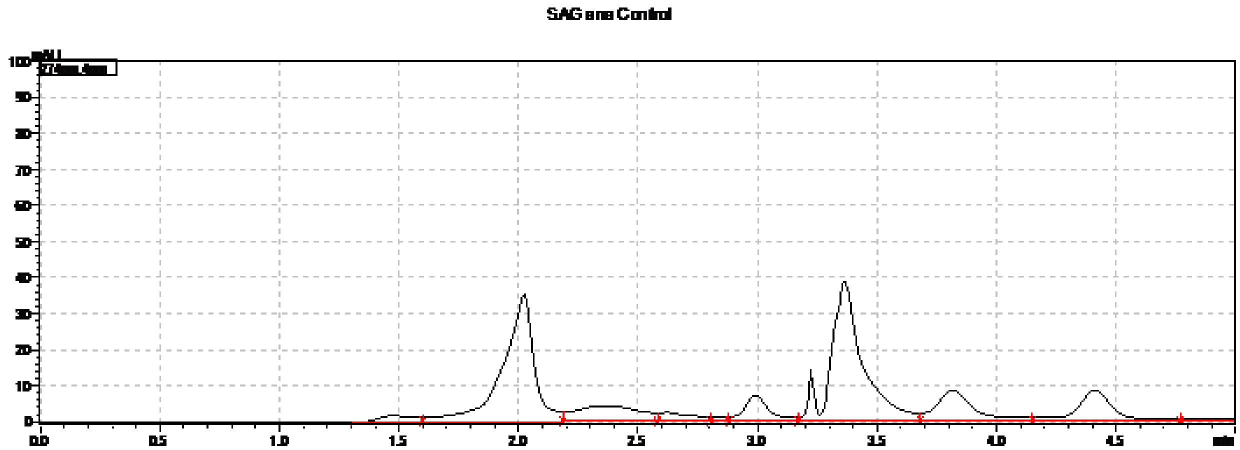

Figure S7e. HPLC trace of control strain BL21(DE3)/pET28a/pET21c.

References:

[1] L.M. Guzman, D. Belin, M.J. Carson, and J. Beckwith, Tight regulation, modulation, and high-level expression by vectors containing the arabinose PBAD promoter. J Bacteriol 177 (1995) 4121-30.

[2] M.K. Ahmadi, L. Fang, N. Moscatello, and B.A. Pfeifer, E. coli metabolic engineering for gram scale production of a plant-based anti-inflammatory agent. Metab. Eng. 38 (2016) 382-388.

[3] G. Zhang, Y. Li, L. Fang, and B.A. Pfeifer, Tailoring pathway modularity in the biosynthesis of erythromycin analogs heterologously engineered in E. coli. Sci. Adv. 1 (2015) e1500077/1-e1500077/8.

[4] T. Baba, T. Ara, M. Hasegawa, Y. Takai, Y. Okumura, M. Baba, K.A. Datsenko, M. Tomita, B.L. Wanner, and H. Mori, Construction of Escherichia coli K-12 in-frame, single-gene knockout mutants: the Keio collection. Mol Syst Biol 2 (2006) 2006 0008.
